# Combined Oral Administration of GABA and DPP-4 Inhibitor Prevents Beta Cell Damage and Promotes Beta Cell Regeneration in Mice

**DOI:** 10.3389/fphar.2017.00362

**Published:** 2017-06-20

**Authors:** Wenjuan Liu, Dong Ok Son, Harry K. Lau, Yinghui Zhou, Gerald J. Prud’homme, Tianru Jin, Qinghua Wang

**Affiliations:** ^1^Department of Endocrinology and Metabolism, Huashan Hospital, Fudan UniversityShanghai, China; ^2^Division of Endocrinology and Metabolism, The Keenan Research Centre in the Li Ka Shing Knowledge Institute, St. Michael’s Hospital, TorontoON, Canada; ^3^Department of Laboratory Medicine and Pathobiology, Keenan Research Centre for Biomedical Science, St. Michael’s Hospital, TorontoON, Canada; ^4^Division of Advanced Diagnostics, Toronto General Research Institutes, University Health Network, TorontoON, Canada; ^5^Institute of Medical Science, University of Toronto, TorontoON, Canada; ^6^Department of Physiology, University of Toronto, TorontoON, Canada; ^7^Department of Medicine, University of Toronto, TorontoON, Canada

**Keywords:** GABA, sitagliptin, streptozotocin, beta cell, diabetes

## Abstract

γ-aminobutyric acid (GABA) or glucagon-like peptide-1 based drugs, such as sitagliptin (a dipeptidyl peptidase-4 inhibitor), were shown to induce beta cell regenerative effects in various diabetic mouse models. We propose that their combined administration can bring forth an additive therapeutic effect. We tested this hypothesis in a multiple low-dose streptozotocin (STZ)-induced beta cell injury mouse model (MDSD). Male C57BL/6J mice were assigned randomly into four groups: non-treatment diabetic control, GABA, sitagliptin, or GABA plus sitagliptin. Oral drug administration was initiated 1 week before STZ injection and maintained for 6 weeks. GABA or sitagliptin administration decreased ambient blood glucose levels and improved the glucose excursion rate. This was associated with elevated plasma insulin and reduced plasma glucagon levels. Importantly, combined use of GABA and sitagliptin significantly enhanced these effects as compared with each of the monotherapies. An additive effect on reducing water consumption was also observed. Immunohistochemical analyses revealed that combined GABA and sitagliptin therapy was superior in increasing beta cell mass, associated with increased small-size islet numbers, Ki67^+^ and PDX-1^+^ beta cell counts; and reduced Tunel^+^ beta cell counts. Thus, beta cell proliferation was increased, whereas apoptosis was reduced. We also noticed a suppressive effect of GABA or sitagliptin on alpha cell mass, which was not significantly altered by combining the two agents. Although either GABA or sitagliptin administration delays the onset of MDSD, our study indicates that combined use of them produces superior therapeutic outcomes. This is likely due to an amelioration of beta cell proliferation and a decrease of beta cell apoptosis.

## Introduction

Type 1 diabetes is characterized by extensive beta cell loss as a result of apoptosis and lack of regeneration. Islet transplantation has been an ultimate treatment for subjects with severe T1D, but its application is limited due to the lack of donors and the need for intense immunosuppression (Ricordi and Strom, 2004; Shapiro, 2012; Sharples et al., 2016). Thus, it is urgent to develop novel therapeutic approaches that can increase the survival and proliferation of endogenous pancreatic beta cells.

During the past two decades, intensive investigation of the incretin hormone GLP-1 has led to the development of two categories of novel therapeutic agents for T2D: GLP-1 agonists and DPP-4 inhibitors (Lee and Jun, 2014; Holst and Madsbad, 2016; Tian and Jin, 2016). The drugs in the latter category, with sitagliptin as an example, prevent the degradation of GLP-1 and another incretin GIP, and hence elevate endogenous incretin levels. In addition to functioning as incretins, GLP-1 as well as GLP-1 based drugs were shown to promote beta cell expansion in various mouse models (Takeda et al., 2012; Drucker, 2013; Nie et al., 2013). Although GLP-1-based drugs were shown to ameliorate T2D (Wang and Brubaker, 2002; Buteau et al., 2003), they showed very marginal therapeutic effects for T1D subjects in both humans and rodent models, possibly due to their restricted immune regulatory effects (Hadjiyanni et al., 2008; Pettus et al., 2013; Wan et al., 2015).

γ-aminobutyric acid, initially identified as an inhibitory neurotransmitter, plays an important role in the regulation of islet cell function and glucose homeostasis (Xu et al., 2006; Soltani et al., 2011; Purwana et al., 2014). We reported that oral administration of GABA prevented and partially reversed T1D in rodent models (Soltani et al., 2011). The preventive and therapeutic effects of GABA in T1D mice were associated with beta cell mass expansion (Soltani et al., 2011; Wan et al., 2015).

Here, we tested the potential additive therapeutic effects of GLP-1-based drug sitagliptin and GABA in a model of multiple low-dose STZ-induced diabetes (MDSD) in C57BL/6J mice. Our aim was the development of a novel and entirely oral therapeutic strategy for T1D. We report that the combined administration of these two agents has an additive therapeutic effect, as compared to the respective monotherapies.

## Materials and Methods

### Animal Handling

**FIGURE 1:**
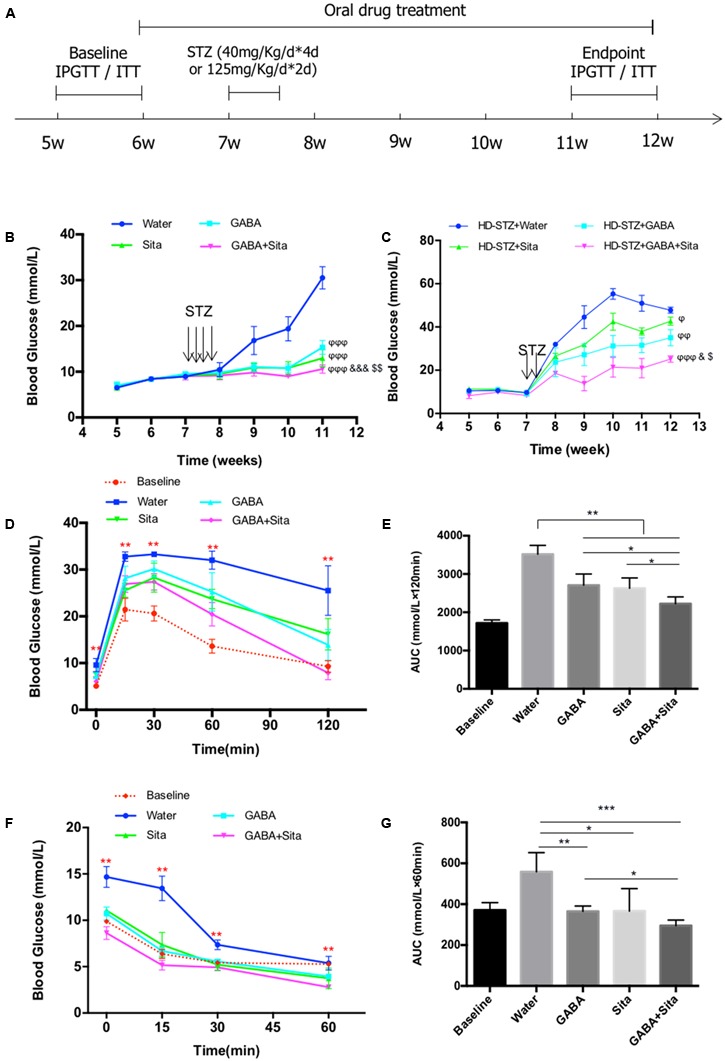
Combined use of GABA and sitagliptin in STZ-induced T1D mouse model generates additive effects on improving glucose tolerance. **(A)** A chart shows the mouse experiment design. **(B)** Longitudinal blood glucose levels in the four groups of mice defined as Water (diabetic control), GABA (GABA treatment), Sita (sitagliptin treatment), and GABA+Sita (GABA plus sitagliptin treatment). **(C)** Longitudinal blood glucose levels in the four groups of mice defined as HD-STZ+Water (diabetic control), HD-STZ+GABA (GABA treatment), HD-STZ+Sita (sitagliptin treatment), and HD-STZ+GABA+Sita (GABA plus sitagliptin treatment). **(D)** IPGTT performed at 5-weeks-old (defined as baseline) as well as at 11-weeks-old. **(E)** Area under curve (AUC) for **(D)**. **(F)** IPITT performed at 5-weeks-old (defined as baseline) as well as at 11-weeks-old. **(G)** Area under curve (AUC) for **(F)**. For **(D–G)**, *n* = 20 for the baseline and *n* = 5 for each of the four groups of mice. Data are mean ± SD. ^∗^*P* < 0.05, ^∗∗^*P* < 0.01. ^ϕ^*P* < 0.05, ^ϕϕ^*P* < 0.01, ^ϕϕϕ^*P* < 0.001 vs. diabetic control group; ^&^*P* < 0.05, ^&&&^*P* < 0.001 vs. GABA treated group; ^$^*P* < 0.05, ^$$^*P* < 0.01 vs. sitagliptin treated group.

Male C57BL/6J mice (4-week-old, approximately 17–18 g) purchased from Jackson Laboratories (Bar Harbor, ME, United States) were housed in a specific pathogen-free animal vivarium at St. Michael’s Hospital, and maintained on a 12-h light-dark cycle, with free access to standard rodent chow and water. For low-dose STZ induced diabetic mouse model, 20 mice were randomly assigned into four groups after 1-week of adaptive housing: non-treatment diabetic control group (Water), GABA treatment group (GABA), sitagliptin treatment group (Sita), and GABA plus sitagliptin group (GABA+Sita) (**Figure 1A**). For high-dose STZ induced diabetic mouse model, 20 mice were also randomly assigned into four groups: non-treatment diabetic control group (HD-STZ+Water), GABA treatment group (HD-STZ+GABA), sitagliptin treatment group (HD-STZ+Sita), and GABA plus sitagliptin group (HD-STZ+GABA+Sita) (**Supplementary Figure S1A**). GABA (Sigma–Aldrich, St. Louis, MO, United States, 6 mg/mL in drinking water), or sitagliptin (Merck Inc., Montreal, ON, Canada, 0.4 mg/L in drinking water), or GABA and sitagliptin were orally administrated at the age of 6 weeks and maintained during the treatment course of 6 weeks. At the age of 7 weeks, all mice received STZ (Sigma–Aldrich, 40 mg/kg for 4 consecutive days or 125 mg/kg for 2 consecutive days) via injection, as we have previously described (Soltani et al., 2011). Body weights and blood glucose levels were measured twice a week; metabolic cages were used before the termination of the experiments (Soltani et al., 2011). Blood samples were collected before treatment and sacrifice. During blood sample collection, diprotin A (Sigma–Aldrich) and aprotinin (Bioshop, Burlington, ON, Canada) were added to inhibit the degradation of GLP-1 and glucagon (Farngren et al., 2014). All animal experiments were conducted in accordance with the guidelines put forth by the Canadian Council on Animal Care and were approved by the University of Toronto Animal Care Committee.

### Blood Glucose Level Determinations, Intraperitoneal Glucose Tolerance Test (IPGTT), and Intraperitoneal Insulin Tolerance Test (IPITT)

Non-fasting blood glucose levels were measured with mouse tail blood using a One Touch Basic glucometer (LifeScan Inc., Burnaby, BC, Canada) or glucose assay kit (Abcam, Cambridge, United Kingdom). Glucose tolerance and insulin sensitivity were evaluated by IPGTT and IPITT at 5–6 weeks old (as baseline) and at 11–12 weeks old (after drug intervention). Mice were fasted for 15 h for IPGTT and 6 h for IPITT, as we described previously (Ip et al., 2015).

### Pancreatic Tissue Preparation, Immunohistochemistry (IHC), and Islet Beta Cell and Alpha Cell Mass Analyses

Pancreases were paraffin embedded and prepared for histological analysis (Wang and Brubaker, 2002). Briefly, freshly isolated pancreas was cut into 8–10 segments followed by formaldehyde fixation. All pancreatic pieces were embedded in paraffin after being dehydrated in ethanol and cleaned with xylene. Insulin and glucagon dual staining were performed on tissue sections (5 μm) by using guinea pig anti-insulin and rabbit anti-glucagon antibodies (1:1000; DAKO, Burlington, ON, Canada); then detected with fluorescent (Cy3- and FITC- conjugated IgG) or biotinylated secondary antibodies (1:200; Abcam, Cambridge, United Kingdom). For IHC staining, samples were incubated with avidin-biotin-peroxidase complex (Vector Laboratories, Burlington, ON, Canada) before staining with DAB (Vector Laboratories) or Fuchsin red (DAKO) and subsequent hematoxylin counterstaining. Entire pancreatic images were scanned and viewed with NanoZoomer (Hamamatsu, Hamamatsu-shi, Shizuoka-ken, Japan) and analyzed by using the ImageScope program (Aperio Technologies, Vista, CA, United States) (Purwana et al., 2014). Total alpha and beta cell mass were determined by the product of cross-sectional alpha and beta cell area over total tissue area and the weight of pancreatic tissue before fixation.

### Measurement of Beta Cell Replication and Apoptosis

Proliferative beta cells were detected in the pancreatic sections by double immunofluorescence staining with guinea pig anti-insulin (1:1000; DAKO), rabbit anti-Ki67 (1:200; Thermo Fisher, Burlington, ON, Canada) antibodies, and relevant secondary antibodies (1:1000; Abcam). Regenerative beta cells were detected by double immunofluorescence staining with guinea pig anti-insulin (1:1000; DAKO), rabbit anti-PDX-1 (1:400; Cell Signaling Technology, Danvers, MA, United States) antibodies, and relevant secondary antibodies (1:200; Jackson ImmunoResearch Laboratories, West Grove, PA, United States). Apoptotic beta cells were also identified in pancreatic sections with insulin and terminal deoxynucleotidyl transferase dUTP nick end labeling (Tunel) labeling (TMR red, Roche, Mississauga, ON, Canada) (Wang and Brubaker, 2002; Robertson et al., 2008). Results are expressed as the percentage of Ki67^+^, PDX-1^+^, or Tunel^+^ beta cells. All immunofluorescent images were captured by an Olympus upright BX50 fluorescence microscope (Olympus, Richmond Hill, ON, Canada) at ×40 magnification.

### Plasma Insulin, Glucagon, and Active GLP-1 Measurement

Plasma insulin, glucagon, and active GLP-1 concentrations were measured using an ultra-sensitive mouse insulin ELISA kit (Crystal Chemical Inc., Wakefield, MA, United States), a glucagon RIA kit (Millipore, Etobicoke, ON, Canada), and a high sensitivity GLP-1 active chemiluminescent ELISA Kit (Millipore), according to the manufacturer’s instructions (Chiang et al., 2014).

### Statistical Analysis

All data were expressed as mean ± SD for four independent experimental groups. Statistical analysis was performed using SPSS for Mac Ver. 20.0 (SPSS, Inc., Chicago, IL, United States). All graphs were made using the Prism program (GraphPad, San Diego, CA, United States). The significance (*P* < 0.05) among different groups was evaluated using one-way ANOVA followed by the Tukey test.

## Results

### Combined Use of GABA and Sitagliptin in MDSD Generates Additive Effect on Glucose Disposal

To assess the potential additive effect of combined use of GABA and sitagliptin in preventing hyperglycemia, we administrated GABA, sitagliptin, or GABA plus sitagliptin orally 1 week before STZ injection (**Figure 1A**). Two weeks after STZ injection, the control diabetic mice developed serious hyperglycemia (16.8 ± 3.1 mM) (**Figure 1B**). Mice in the three treatment groups, however, showed significantly lower ambient blood glucose levels (<11 mM). At 2 weeks after STZ injection and thereafter, mice from the GABA+sitagliptin group showed lower blood glucose levels when compared with those that received a monotherapy. Whereas the non-treated control mice developed severe hyperglycemia (30.5 ± 0.9 mM) after a 6-weeks period, mice receiving GABA or sitagliptin monotherapy showed significantly lower glycemic levels (15.3 ± 0.5 mM and 13.0 ± 0.6 mM). Moreover, blood glucose level in the GABA+sitagliptin group was at an even lower range (10.6 ± 0.3 mM, *P* < 0.001 between GABA+Sita group and GABA group; *P* < 0.01 between GABA+Sita group and Sita group) (**Figure 1B**).

To further demonstrate that the combined therapy has a superior effect on lowering glucose levels, we also examined high-dose STZ injection, and this revealed larger and more significant differences between the combined therapy group and monotherapy groups (**Figure 1C**). After 6-weeks treatment, the glucose levels of HD-STZ+Water, HD-STZ+GABA, HD-STZ+Sita, and HD-STZ+GABA+Sita groups are 51.0 ± 8.3, 31.6 ± 3.4, 37.9 ± 3.4, and 21.0 ± 7.7 mM, respectively (*P* < 0.05 between HD-STZ+GABA+Sita group and HD-STZ+GABA group; *P* < 0.05 between HD-STZ+GABA+Sita group and HD-STZ+Sita group).

To test whether this combined therapy leads to a better glucose and insulin challenge response, IPGTT and IPITT were performed before (indicated as baseline in **Figures 1D–G**) and 5 weeks after the drug treatment. As shown in **Figures 1D,E**, GABA or sitagliptin monotherapy partially improved glucose tolerance while the combined therapy improved the tolerance even further. **Figures 1F,G** shows that insulin sensitivity is increased by either GABA or sitagliptin monotherapy, while combined therapy tended to increase this even further, albeit without statistical significance.

We then determined circulating insulin and glucagon levels in each of the four groups of mice before and after drug treatment. Both GABA and sitagliptin monotherapies increased plasma insulin levels and decrease plasma glucagon levels (**Table 1**). An additive effect on the elevation of plasma insulin levels but not on the repression of plasma glucagon levels was observed with the combined therapy. As anticipated, plasma GLP-1 levels in mice that received sitagliptin or GABA plus sitagliptin were significantly elevated (**Table 1**).

**Table 1:** Combined use of GABA and sitagliptin elevates plasma insulin and GLP-1 levels, while decreases plasma glucagon levels.

	Baseline	Water	GABA	Sita	GABA+Sita	*F*-values	*P*-values
Insulin (ng/ml)	0.67 ± 0.08	0.30 ± 0.02	0.52 ± 0.14ˆ*	0.44 ± 0.10ˆ*#	0.77 ± 0.22ˆ**	8.43	<0.001
Glucagon (pg/ml)	175.0 ± 21.9	383.8 ± 37.5	212.7 ± 14.5ˆ**	227.8 ± 14.8ˆ**#	199.9 ± 17.1ˆ**	54.89	<0.0001
GLP-1 (pmol/L)	5.26 ± 1.31	2.13 ± 0.43	2.94 ± 0.76	5.10 ± 0.45ˆ**##	6.93 ± 0.29ˆ***	19.28	<0.001

*Data are Mean ± SD. ^∗^P < 0.05, ^∗∗^P < 0.01, ^∗∗∗^P < 0.001 vs. diabetic control group (Water group); ^#^P < 0.05, ^##^P < 0.01 vs. GABA+sitagliptin treated group.*

### Combined Use of GABA and Sitagliptin Improves Metabolic Status in the T1D Mouse Model

**FIGURE 2:**
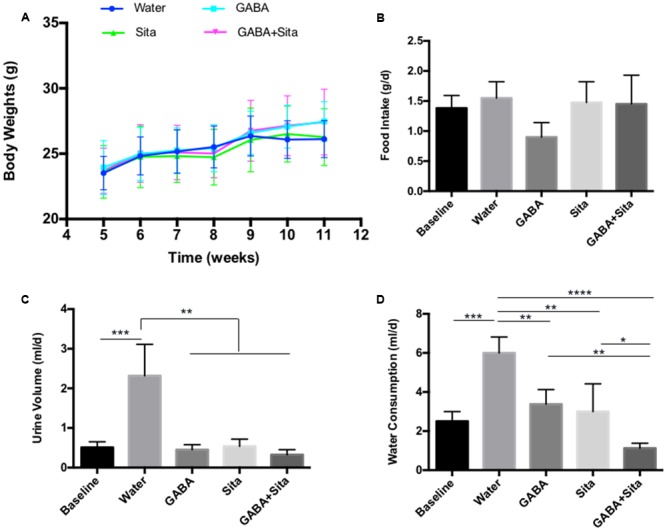
Combined use of GABA and sitagliptin improves metabolic status in STZ-induced T1D mice **(A)** Body weight measurement. **(B)** 24-h food intake. **(D)** 24-h water consumption. **(C)** 24-h urine volume. For **(B–D)**, the data were obtained by putting each individual mouse into a metabolic cage. *n* = 5 for each of the experimental groups. Data are mean ± SD. ^∗^*P* < 0.05, ^∗∗^*P* < 0.01.

Expected therapeutic effects on T1D include the reduction of water consumption and urine volume, as well as an improvement in body weight gain. Although we did not find any effects of GABA, sitagliptin, or combined therapy on body weight changes or food intake (**Figures 2A,B**), we observed a repression of water consumption in mice that received GABA or sitagliptin treatment (**Figure 2D**). Importantly, combined therapy decreased water consumption even further (**Figure 2D**). **Figure 2C** shows that the daily urine volume was lower in mice of the GABA group, the sitagliptin group, and the GABA+sitagliptin group when compared with the non-treatment diabetic controls.

In this MDSD model, body weight differences among the four groups of mice were very marginal. Nevertheless, we have also tested the therapeutic effect of GABA, sitagliptin, and GABA+sitagliptin in mice that received a large-dose of STZ (**Supplementary Figure S1A**). As shown in **Supplementary Figure S1B**, each of the three treatments increased the body weight of the mice with time.

### Combined Use of GABA and Sitagliptin Generates Additive Effects on Increasing Beta Cell Mass

**FIGURE 3:**
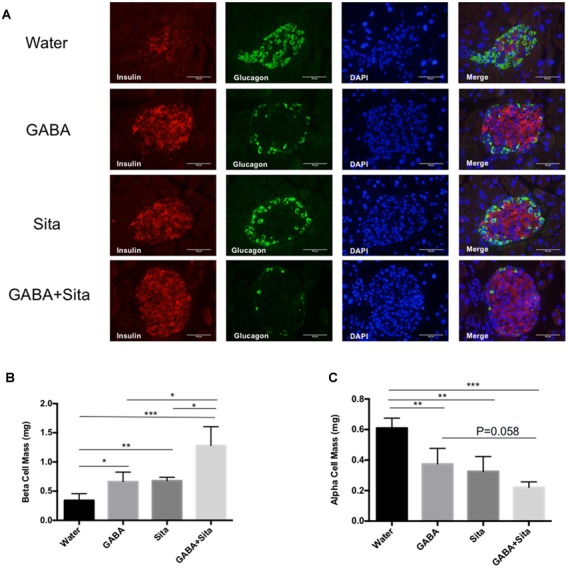
Combined use of GABA and sitagliptin generates additive effects on increasing beta cell mass. **(A)** Representative immunofluorescence images of pancreatic islets, showing insulin staining (red) and glucagon staining (green) in the four groups of mice. **(B,C)** Pancreatic beta cell mass **(B)** and alpha cell mass **(C)** in the four groups of mice. *n* = 5 per defined group. Data are mean ± SD. ^∗^*P* < 0.05, ^∗∗^*P* < 0.01, ^∗∗∗^*P* < 0.001.

To initiate the exploration on mechanisms underlying the additive improvement with the combined therapy, we determined beta cell and alpha cell mass in the four groups of mice. As shown in **Figure 3A**, STZ injection destroyed nearly all beta cells, while the residual islets contained mostly alpha cells. GABA or sitagliptin monotherapy partially mitigated such changes, while the combined therapy altered the changes the most. **Figures 3B,C** shows that either one of the monotherapies increased beta cell mass and decreased alpha cell mass. Importantly, combined therapy increased beta cell mass by nearly twofold when compared with either of the mono-therapeutic approaches, and generated a trend in further reducing alpha cell mass.

### Combined Use of GABA and Sitagliptin Generates an Additive Effect on Increasing Beta Cell Proliferation and Reducing Beta Cell Apoptosis

**FIGURE 4:**
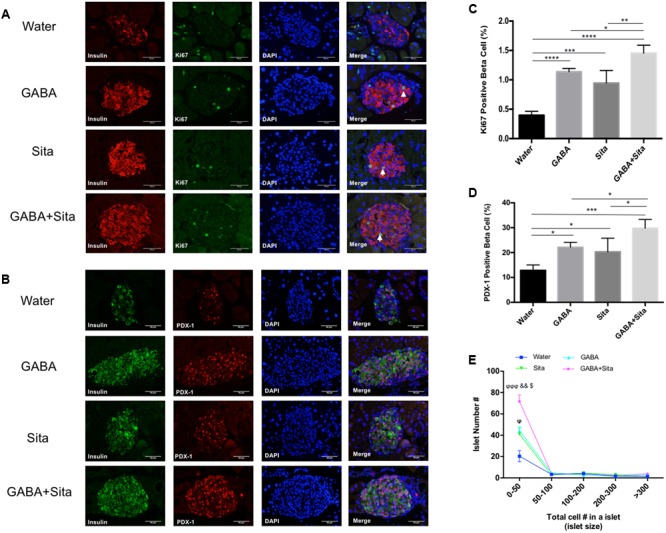
Combined use of GABA and sitagliptin generates an additive effect on increasing beta cell proliferation and regeneration. **(A)** Representative immunofluorescence images of pancreatic islets, showing Ki67^+^ (green) and insulin^+^ (red) cells in the four groups of mice. **(B)** Representative immunostaining images of pancreatic islets, showing PDX-1^+^ (red) and insulin^+^ (green) cells. **(C,D)** Calculation of percent of Ki67^+^ cells **(C)** and PDX-1^+^ cells **(D)** in the four groups of mice. **(E)** Islet number for different size per section area in four groups of mice. *n* = 5 for each group of mice. Data are mean ± SD. ^∗^*P* < 0.05, ^∗∗^*P* < 0.01, ^∗∗∗^*P* < 0.001, ^∗∗∗∗^*P* < 0.0001; ^ϕ^*P* < 0.05, ^ϕϕϕ^*P* < 0.001 vs. diabetic control group; ^&&^*P* < 0.01 vs. GABA treated group; ^$^*P* < 0.05 vs. sitagliptin treated group.

As GABA or GLP-1 can promote mouse beta cell survival and replication (Wang and Brubaker, 2002; Soltani et al., 2011), we assessed whether combined therapy could generate an additive effect in this MDSD model. We used a Ki67/insulin dual immunofluorescence staining approach to determine the beta cell replication rates. In the MDSD mice that received no drug treatment, the rate of beta cell proliferation was 0.40 ± 0.03%, consistent with our previous report under the similar experimental conditions (Soltani et al., 2011). In contrast, the rates of beta cell proliferation in mice that received GABA or sitagliptin treatment were ∼1.12 and 0.94%, respectively (**Figures 4A,C**). Moreover, mice that received the combined therapy showed further increase in the rate of beta cell proliferation (∼1.45%).

To further elucidate the underlying mechanism of beta cell replication under our treatment conditions, we performed dual staining for insulin and PDX-1, one of the key beta cell developmental markers. Consistent with the Ki67 results, the PDX-1^+^insulin^+^ cell counts are significantly increased in GABA (22.14 ± 1.97%) and sitagliptin (20.32 ± 5.45%) treated groups compared with non-treated diabetic mice (12.84 ± 2.17%); while combined therapy increased this even more (29.83 ± 3.49%) (**Figures 4B,D**). In addition, we also demonstrated that the small-size islet numbers (beta cell count less than 50) are significantly higher in the combined therapy group (**Figure 4E**), which might partially explain the pro-regenerative effects of combined use of GABA and sitagliptin.

**FIGURE 5:**
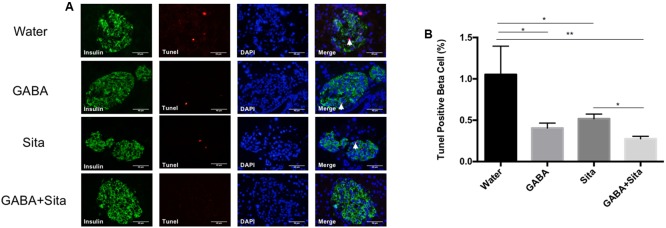
Combined use of GABA and sitagliptin generates an additive effect on reducing beta cell apoptosis. **(A)** Representative immunostaining images of pancreatic islets, showing Tunel^+^ (red) and insulin^+^ (green) cells. **(B)** Calculation of percent of Tunel^+^ cells in the four groups of mice. *n* = 5 for each group of mice. Data are mean ± SD. ^∗^*P* < 0.05, ^∗∗^*P* < 0.01.

The Tunel and insulin double immunostaining method was then utilized to determine beta cell apoptosis. As shown in **Figures 5A,B**, GABA or sitagliptin monotherapy decreased the Tunel positive beta cells, while GABA+sitagliptin treatment generated an additive effect on reducing beta cell apoptosis. As previously reported by our group and others (Prud’homme and Chang, 1999; Maksimovic-Ivanic et al., 2002; Soltani et al., 2007, 2011), beta cell apoptosis is a slower process in an MDSD model compared with high-dose STZ-induced diabetes. Notably, beta cell death in MDSD is largely dependent on inflammation (insulitis), and apoptosis is still apparent weeks after STZ administration, as shown by our current results. Thus, our Tunel staining results in MDSD suggest that combined GABA and sitagliptin treatment exerts superior and long-term protection against beta cell death.

## Discussion

As there is no single therapy that restores recommended glycemic control in the majority of T1D subjects, we investigated a combined therapy with a current drug (sitagliptin) and a potential future drug (GABA) to accomplish this goal. We found that such combined therapy resulted in superior therapeutic outcomes in MDSD, including the prevention of hyperglycemia, the improvement of glucose tolerance, and the reduction of water consumption. Importantly, combined therapy augmented beta cell proliferation and regeneration, and concurrently improved protection against beta cell apoptosis. Combined therapy only marginally altered body weight in MDSD, but weight was ameliorated in mice with high-dose STZ-induced diabetes.

Glucagon-like peptide-1 improves glucose homeostasis via a number of defined mechanisms including its incretin effect on pancreatic beta cells, shared with another incretin hormone GIP (Chiang et al., 2012; Tian and Jin, 2016). In pancreatic islets, GLP-1 also inhibits glucagon secretion, promotes beta cell proliferation and protects beta cell from apoptosis (Brubaker and Drucker, 2004; Yusta et al., 2006; Drucker, 2007, 2013; Wang et al., 2007; Shao et al., 2013). Interestingly, we found that the GLP-1 levels in non-treated MDSD diabetic mice are lower at 12-weeks old compared to the baseline (6-weeks old), which is consistent with several other animal studies. In db/db mice, it was found that the plasma active GLP-1 levels were decreased compared to normal mice, while DPP-4 inhibitor vildagliptin could reverse this change (Wu et al., 2015). The plasma GLP-1 level in our STZ-induced diabetic mice was ∼2 pmmol/L, which was further supported by previous reports using STZ-induced diabetic mice or NOD mice (Kim et al., 2008, 2009). However, the reason for the GLP-1 decline in our current study, and the previous work of others, is not known and requires further investigation.

Dipeptidyl peptidase-4 inhibitors, such as sitagliptin, prevent the degradation of GLP-1 and GIP and hence increase endogenous incretin hormone levels (Kieffer et al., 1995; Tian and Jin, 2016). Although GLP-1 analogs and DPP-4 inhibitors have been broadly utilized in T2D treatment, they had minimal beneficial impacts in treating T1D (Rother et al., 2009; Pettus et al., 2013). We chose the DPP-4 inhibitor sitagliptin for this combined therapy study, as it can be orally administrated with GABA (Dobrian et al., 2011). Clinically, oral administration of drugs is advantageous, especially for chronic diseases with long-term treatment. As anticipated, sitagliptin administration indeed increased plasma GLP-1 levels in our mouse model. Nevertheless, as DPP-4 can degrade a number of other peptide hormones including GIP and NPY, further investigations are needed to assess whether other DPP-4 substrates participate in the additive metabolic beneficial effect observed in our combined therapy experiments.

In addition to the increase of beta cell mass with either of the monotherapies or the combined therapy presented in this study, we found that GABA, sitagliptin, or GABA plus sitagliptin reduced alpha cell mass as well as plasma glucagon levels. The reduction of alpha cell mass by GABA treatment in the T1D mouse model is in agreement with our previous study (Soltani et al., 2011), while the reduction of alpha cell mass by sitagliptin or its derivatives was reported previously in both T1D and T2D mouse models (Mu et al., 2006; Takeda et al., 2012). It is important to point out that very recent studies have indicated that GABA induces alpha cell to beta cell transdifferentiation, starting with the conversion of pancreatic duct cells into alpha cells (Ben-Othman et al., 2017; Li et al., 2017). When normal mice at different ages (2.5–10 months) received GABA treatment for 2–3 months, their insulin^+^ as well as glucagon^+^ cell numbers were increased. Thus, during long-term GABA administration, a transient increase in alpha cell mass was observed. In the current study, we assessed both alpha cell mass and plasma glucagon levels 6 weeks after GABA or GABA plus sitagliptin treatment; and did not observe the potential transient increase of alpha cells. However, our treatment period was shorter than in studies showing transdifferentiation and related changes in the alpha cell population, which might explain this difference in our results.

Previous studies have shown that GLP-1, through binding to its receptor GLP-1R (De Leon et al., 2003), promotes beta cell replication and prevent beta cell apoptosis via the activation of P13K/Akt and CREB-IRS2 signaling pathways (Wang and Brubaker, 2002; Wang et al., 2004; Whalley et al., 2011). These two pathways were also shown to mediate the function of GABA in maintaining beta cell mass (Soltani et al., 2011; Purwana et al., 2014). We show here that GABA and sitagliptin combined therapy further increased beta cell mass, associated with increased Ki67^+^ or PDX-1^+^ beta cell number and reduced Tunel^+^ beta cell number. Extensive further *in vitro* and *in vivo* examinations are needed to address whether such additive effects are simply due to the additive stimulation of these two signaling cascades, or due to some yet to be identified underlying mechanisms. Nevertheless, cross-talk between GABA and GLP-1 signaling cascades have been suggested at different levels. In rat hippocampal CA3 pyramidal neurons, both native GLP-1 and its agonist exendin-4 were shown to enhance GABA_A_ receptor-mediated synaptic and tonic currents (Korol et al., 2015). In rat islets, GLP-1 treatment leads to increased GABA production (Wang et al., 2007). Furthermore, GABA treatment was shown to increase GLP-1 production in a mouse gut endocrine L cell line model (Gameiro et al., 2005).

## Conclusion

Our observations suggest that combined use of GABA and sitagliptin in T1D treatment is feasible, leading to a delay in the onset of STZ-induced T1D and additive therapeutic effects on metabolic profiles. As sitagliptin and other DPP-4 inhibitors can be orally administrated, new drugs have been developed by combining a given DPP-4 inhibitor with metformin or pioglitazone, known as Janumet and Oseni, respectively (Jin and Weng, 2016). GABA can also be orally administered, and this is an important clinical advantage. Further pre-clinical and clinical trials are warranted to test the efficacy and toxicity of combined use of GABA and sitagliptin in T1D mouse models and T1D human subjects.

## Data Availability

The data that support the findings of this study are available from the corresponding author upon reasonable request.

## Author Contributions

QW is the guarantor of the study and, as such, has full access to all the data in the study and takes responsibility for the integrity of the data and the accuracy of the data analysis. WL, DS, HL, and YZ have conducted the study and contributed the data for the manuscript. QW designed the experiments. WL and DS have drafted the manuscript. QW, TJ, and GP have edited the manuscript. All authors approved the version for the submission.

## Conflict of Interest Statement

The authors declare that the research was conducted in the absence of any commercial or financial relationships that could be construed as a potential conflict of interest.

## Supplementary Material

FIGURE S1 GABA, sitagliptin, or GABA+sitagliptin in large-dose of STZ-induced T1D mouse model generates protective effects on the improving the metabolic status. **(A)** A chart shows the mouse experimental design. **(B)** Longitudinal body weight measurement in the four groups of mice defined as HD-STZ+Water (diabetic control), HD-STZ+GABA (GABA treatment), HD-STZ+Sita (sitagliptin treatment), and HD-STZ+GABA+Sita (GABA plus sitagliptin treatment). For **(B)**, *n* = 20 for the baseline and *n* = 5 for each of the four groups of mice. Data are mean ± SD.

## Abbreviations

DPP-4: dipeptidyl peptidase-4
ELISA: enzyme-linked immunosorbent assay
GABA: γ-aminobutyric acid
GIP: gastric inhibitory polypeptide
GLP-1: glucagon-like peptide-1
IHC: immunohistochemistry
IPGTT: intraperitoneal glucose tolerance test
IPITT: intraperitoneal insulin tolerance test
MDSD: multiple low-dose streptozotocin-induced beta cell injury mouse model
NPY: neuropeptide Y
PDX-1: pancreatic and duodenal homeobox 1
RIA: radioimmunoassay
STZ: streptozotocin
T1D: type 1 diabetes
T2D: type 2 diabetes
Tunel: terminal deoxynucleotidyl transferase dUTP nick end labeling.
