# Supplementary figures and images for: Combined Oral Administration of GABA and DPP-4 Inhibitor Prevents Beta Cell Damage and Promotes Beta Cell Regeneration in Mice
Source: Front Pharmacol. 2017 Jun 20;8:362. doi: 10.3389/fphar.2017.00362 (PMC5476705; doi:10.3389/fphar.2017.00362)

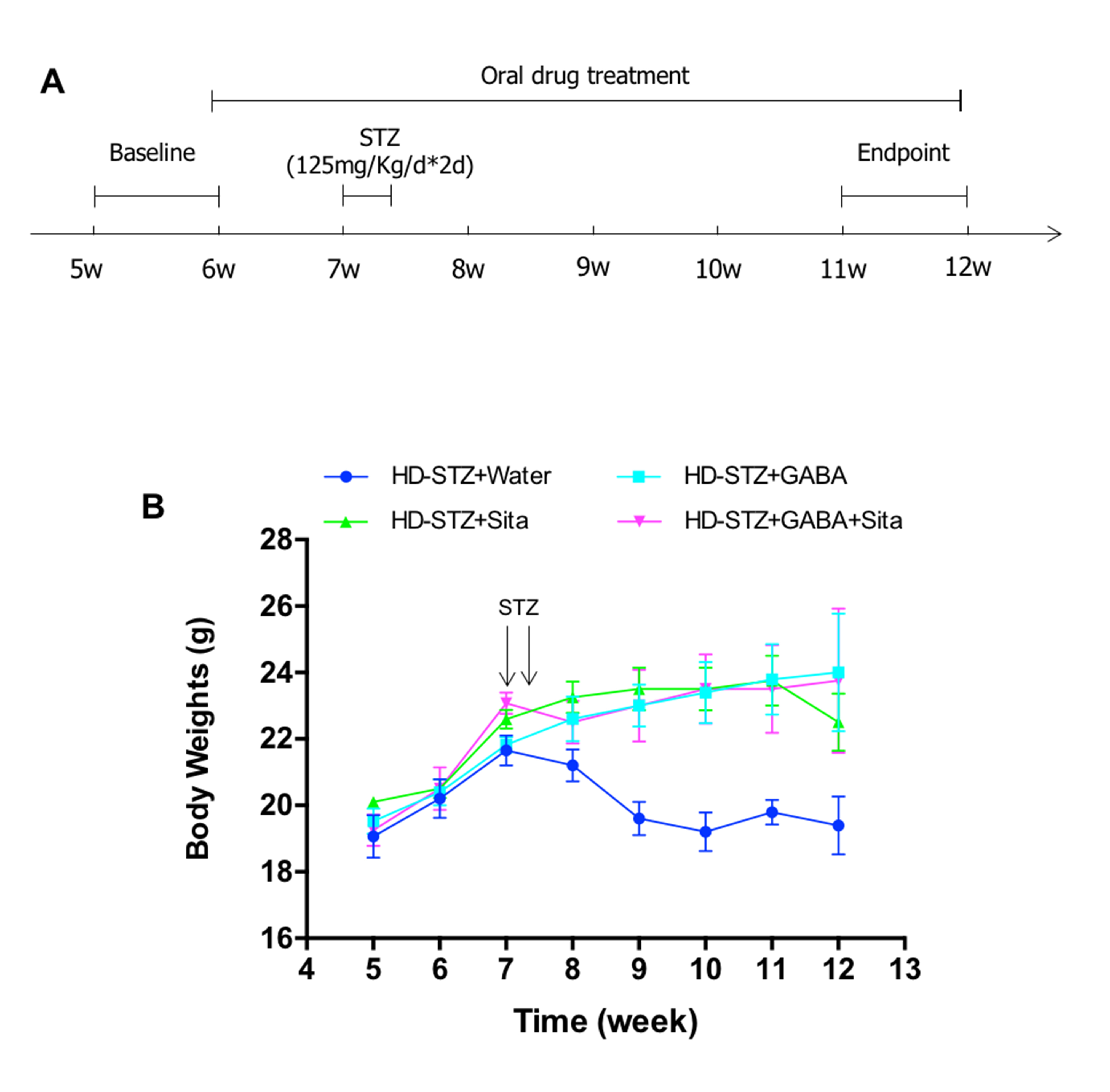

Supplement: FIGURE S1 — GABA, sitagliptin, or GABA+sitagliptin in large-dose of STZ-induced T1D mouse model generates protective effects on the improving the metabolic status. (A) A chart shows the mouse experimental design. (B) Longitudinal body weight measurement in the four groups of mice defined as HD-STZ+Water (diabetic control), HD-STZ+GABA (GABA treatment), HD-STZ+Sita (sitagliptin treatment), and HD-STZ+GABA+Sita (GABA plus sitagliptin treatment). For (B), n = 20 for the baseline and n = 5 for each of the four groups of mice. Data are mean ± SD. [file Image_1.JPEG]
